# Pointwise Structure–Function Analysis of the Ellipsoid Zone in Retinitis Pigmentosa Using an Artificial Intelligence-Assisted OCT and Microperimetry Overlay

**DOI:** 10.1016/j.xops.2025.100889

**Published:** 2025-07-21

**Authors:** Jesse A. Most, An D. Le, Melanie D. Tran, Evan H. Walker, Aneesh Swamy, Dirk-Uwe G. Bartsch, William R. Freeman, Truong Nguyen, Cheolhong An, Shyamanga Borooah

**Affiliations:** 1 Jacobs Retina Center, Shiley Eye Institute, University of California San Diego, La Jolla, California; 2 School of Medicine, University of California San Diego, La Jolla, California; 3 Department of Electrical and Computer Engineering, University of California San Diego, La Jolla, California; 4 Viterbi Family Department of Ophthalmology and Shiley Eye Institute, University of California San Diego, La Jolla, California; 5 University of California San Diego, La Jolla, California

**Keywords:** Retinitis pigmentosa, Ellipsoid zone, Spectral domain optical coherence tomography, Structure–function

## Abstract

**Objective:**

To perform a pointwise structure–function analysis of the ellipsoid zone (EZ) in retinitis pigmentosa (RP) using an artificial intelligence–based overlay to understand EZ structure–function relationships.

**Design:**

A single-center retrospective study.

**Subjects:**

Patients with clinically confirmed RP.

**Methods:**

Same-day spectral-domain OCT (SD-OCT) and microperimetry near-infrared images were overlaid in patients with confirmed RP. Overlay used a coarse alignment artificial intelligence model. Each locus, on a 68-point microperimetry grid spanning the central 20° of the macula, was identified on individual SD-OCT B-scans. Ellipsoid zone structure was graded at each locus on a 3-point scale: grade 0 = EZ not visible; grade 1 = EZ attenuated; grade 2 = EZ normal. Ellipsoid zone grades were correlated with microperimetry sensitivity scores recorded in decibels (dB).

**Main Outcome Measures:**

Correlation of EZ integrity on SD-OCT with microperimetric retinal sensitivity.

**Results:**

Fifty-one eyes from thirty-one patients with RP were included in the analysis, with 60 total overlays, including follow-up studies, resulting in 3985 test loci being graded. Patients had a mean age of 39.4 (32.8–46.0) years, with 41.9% being female. Mean best-corrected visual acuity was 0.166 (0.129–0.203) logarithm of the minimum angle of resolution. The overall mean sensitivity (MS) was 9.72 (7.32–12.12) dB, whereas MS was 6.02 (4.06–7.98) dB for grade 0 loci, 18.36 (16.35–20.36) dB for grade 1 loci, and 20.90 (18.87–22.93) dB for grade 2 loci. Differences in MS were significant between graded groups (*P* < 0.001). Correlation between EZ grade and sensitivity was 0.65 (0.64–0.67), whereas correlation of sensitivity with distance from the fovea was −0.41 (−0.43 to −0.39). Focusing on grade 0 loci, 57.5% had sensitivity scores >0 dB, and 4% had scores ≥20 dB, suggesting that these points had function despite no observable EZ on SD-OCT.

**Conclusions:**

We identified local EZ structure–function incongruencies in RP using a pointwise analysis of structure–function overlay. These loci of interest may be overlooked in analyses that average across the visual field. Preserved photoreceptor function, in the absence of visibly intact EZ, warrants further investigation.

**Financial Disclosure(s):**

Proprietary or commercial disclosure may be found in the Footnotes and Disclosures at the end of this article.

Retinitis pigmentosa (RP) encompasses a group of inherited retinal diseases characterized by progressive photoreceptor degenerations. Mutations linked to over 90 genes have been implicated in this rod–cone dystrophy.^1^^,^^2^ There are estimated to be over 1.5 million people living with RP worldwide.^3^^,^^4^ Although there is genotypic and phenotypic heterogeneity, RP classically presents with nyctalopia followed by the progressive loss of peripheral and eventually central vision.^1^ This results in visual impairment and, in most cases, legal blindness,^5^ often with devastating quality of life implications.^6^

Although there is currently only 1 approved treatment for RP, numerous clinical trials are ongoing, and some show promising results.^7^^,^^8^ To facilitate these trials and provide accurate prognostic information to patients, it is crucial for researchers and clinicians to have the ability to assess and monitor disease progression. For this purpose, RP trials often rely on multimodal imaging.^9^ Spectral-domain OCT (SD-OCT) offers a minimally invasive alternative for monitoring progression with lower repeat variability. This is because predictable structural changes can be observed with photoreceptor loss in RP.^10^ In classical RP, moving outward from the fovea, these changes include a thinning of the retinal outer segment (OS), followed by the outer nuclear layer, and then complete loss of the OS and ellipsoid zone (EZ) band.^1^^,^^10^ Ellipsoid zone integrity has been shown to be a particularly sensitive and reliable marker for tracking RP progression on SD-OCT, and EZ change has been suggested as an endpoint for clinical trials.^9^, ^10^, ^11^, ^12^, ^13^ Prior investigations coregistering OCT and microperimetry have demonstrated a correlation between loss of EZ width and area with reduced visual function as measured by mean sensitivity (MS).^13^ However, sensitivity analyses tend to use global measures of visual function rather than a pointwise approach, which may miss pointwise discrepancies in EZ integrity and function, which may shelter visual function.

The present study aims to complete a pointwise structure–function analysis using recently developed artificial intelligence algorithms^14^ by overlaying near-infrared (NIR) images of SD-OCT scans onto corresponding NIR images from microperimetry studies. This overlay facilitates analysis of sensitivity versus EZ integrity at 68 unique loci across the macula, possibly offering new insights into the structure–function relationship and overall utility of the EZ as a biomarker in RP.

## Methods

This is a retrospective cross-sectional study of patients with RP seen in the inherited retinal disease clinic at the University of California, San Diego between December 1, 2020, and October 31, 2024. The University of California, San Diego Institutional Review Board (Protocol #120516) approved the study for a waiver of informed consent given the retrospective nature of the work. The study adhered to the tenets of the Declaration of Helsinki.

Retinitis pigmentosa patients were initially identified using the inherited retinal disease database at the University of California, San Diego and RP was confirmed by a retinal specialist. Patient age, sex, ethnicity, and best-corrected visual acuity were recorded. Anonymized SD-OCT (Spectralis HRA + OCT, Heidelberg Engineering) and Macular Integrity Assessment (MAIA, CenterVue S.p.A.) microperimetry studies were gathered in pairs for patients who had completed both studies on the same day. Follow-up studies were also included if available. Exclusion criteria included eyes with other retinal pathology, a history of retinal surgery, or intraocular surgery completed within 6 months. Eyes with epiretinal membrane distorting retinal structure or cystoid macular edema were also excluded as potential confounders. Images without clearly visible third-order arterioles on NIR images were deemed to be of poor quality and excluded because this would prevent accurate overlay. Microperimetry studies with fixation losses >30% were also excluded, per the manufacturer cutoff for a reliable test.^15^

Retinal structure was visualized using the HRA + OCT Spectralis System. Spectral-domain OCT scans were captured as 49 B-scans (512 A-scans per B-scan), with 20.0° × 20.0° (6.1 × 6.1 mm) pattern size, 120 μm between scans, and automatic real-time tracking enabled. Image quality scores were above the manufacturer recommendation for clinical use.^16^ Retinal function was measured using MAIA microperimetry, which recorded retinal sensitivity at 68 test loci arranged in an array centered on the fovea. Each locus was separated by 2° at 1°, 3°, 5°, 7°, and 9° from the vertical or horizontal midlines in a similar arrangement to the Humphrey visual field 10-2 testing grid.^17^ Goldman size III stimuli were presented for 200 ms against a background luminance of 1.27 cd/m^2^ using a 4-2 testing strategy, equivalent to mesopic testing. At each locus, the MAIA software provided threshold retina sensitivity in decibels (dB) ranging from a minimum of <0 dB to a maximum of 36 dB. The HRA + OCT Spectralis alignment between the scanning laser ophthalmoscope and OCT path was performed on a regular basis according to the manufacturer’s guidelines to ensure accurate alignment of the OCT path to the scanning laser ophthalmoscope path.^18^ The coalignment was always better than 1/2 pixel.

**Figure 1:**
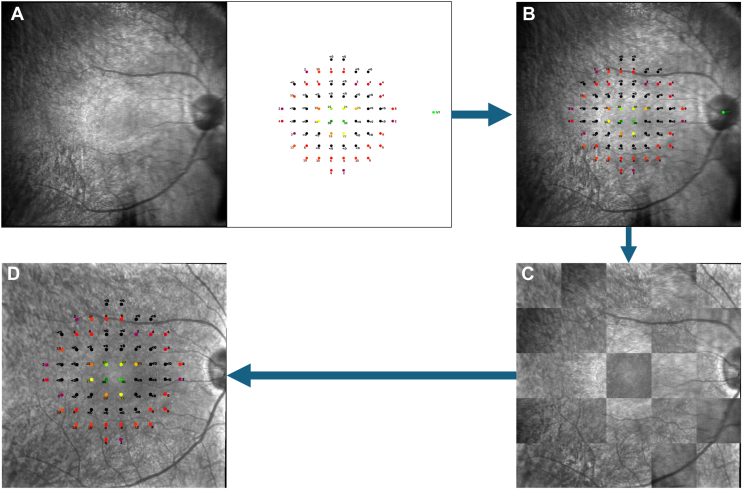
Visualization of SD-OCT and microperimetry alignment. Microperimetry NIR and locus sensitivity map (**A**) were overlaid onto corresponding SD-OCT NIR images (**B**). Checkerboard overlay (**C**) was aligned with SD-OCT NIR, and vessels were segmented to allow identification of key points using the SuperPoint network. A perspective transformation matrix was computed to align the two images based on these key points. The microperimetry sensitivity map was overlaid onto corresponding SD-OCT NIR images (**D**). The same transformation matrix was applied to the sensitivity map to find the location of microperimetry test loci on the corresponding OCT image. SD-OCT = spectral-domain OCT; NIR = near-infrared.

**Figure 2:**
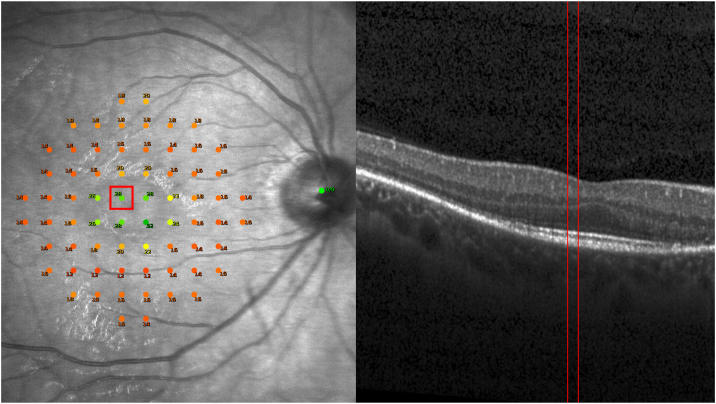
Pointwise identification of a microperimetry locus on SD-OCT. A NIR microperimetry image with a 68-loci grid and respective sensitivity scores (dB) (left). The locus marked in red at left corresponds to the structural location on SD-OCT (right) demarcated by the vertical red borders. SD-OCT = spectral-domain OCT; NIR = near-infrared.

To directly correlate structure with function, NIR images from MAIA microperimetry (850 nm) were overlaid onto NIR images from SD-OCT scans (815 nm). The same artificial intelligence algorithm of our group’s previous work^14^ was used to facilitate this alignment. In short, retinal vessels in both images were segmented, followed by the identification of key vessel features using the SuperPoint neural network.^19^ These features were then used to compute a perspective transformation matrix to align the images, which were initially of different dimensions (SD-OCT NIR: 768 x 768 pixels; MAIA NIR: 1024 x 1024 pixels) (Fig 1). With the alignment complete, individual SD-OCT B-scans were then labeled with borders marking the location of each microperimetry locus (Fig 2). The width of each locus was set at 10 pixels (110 μm) based on the radius of a MAIA locus on NIR (5 pixels) and had borders (1 pixel thick) flanking each side of the graded area. One labeled B-scan was generated per locus based on the closest B-scan, resulting in a maximum of 68 images per eye in total. In cases where microperimetry loci were mapped to locations outside the borders of the OCT NIR raster following alignment, these loci were excluded from analysis, resulting in <68 total images for that eye.

**Figure 3:**
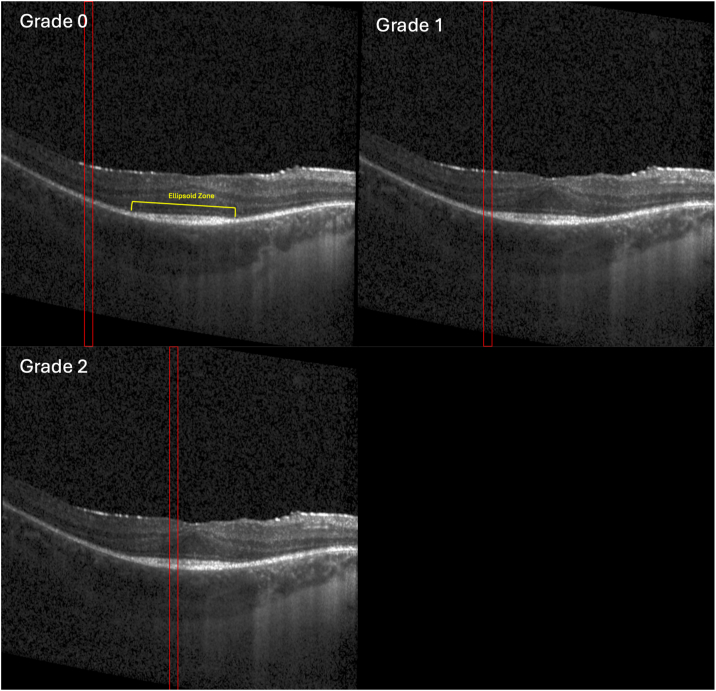
Ellipsoid zone grade examples on SD-OCT. Representative SD-OCT B-scans corresponding to 3 consecutive loci and respective EZ grades. Loci borders (red) are determined via NIR OCT and NIR microperimetry overlay. Estimated EZ marked in yellow (top left). Grade 0 = no EZ visible (top left), grade 1 = attenuated EZ visible (top right), and grade 2 = normal EZ visible (bottom left). SD-OCT = spectral-domain OCT; NIR = near-infrared; EZ = ellipsoid zone.

Individually labeled B-scans were then assessed for EZ layer integrity. A three-point grading scale from 0 to 2, similar to that described by Aizawa et al,^20^ was used to grade the appearance of the EZ: grade 0 = not visible; grade 1 = attenuated; grade 2 = normal (Fig 3). Two graders (J.M. and M.T.) independently graded each image after training with a senior retina specialist (S.B.). For any loci scores with grader disagreement, final grades were adjudicated by a senior retina specialist (S.B.).

Microperimetry sensitivity scores were matched to corresponding EZ grades at each locus. Mean sensitivity was calculated for all loci of grades 0, 1, and 2, respectively. For computations, loci with sensitivity of <0 dB were counted as −1 dB. Demographic and ocular characteristics are presented as count (%) and mean (95% confidence interval) for categorical and continuous parameters, respectively. Eye-level continuous parameters were evaluated using linear mixed-effects models. Mixed models were also utilized to assess mean differences in sensitivity across ordinal EZ scores. Post hoc testing was used to assess pairwise comparisons of mean differences in sensitivity across all EZ score pairs, with Tukey’s adjustment applied for multiple testing. Residuals were evaluated across all mixed-effects models through the inspection of visualization of residuals versus fitted values plots, Q-Q plots, and histograms to assess homoscedasticity and normality. As a preliminary analysis, the Spearman rank nonparametric correlation coefficient was estimated to depict the relationship between the ordinal EZ score and MS, stratified by degrees from the fovea. Further analysis of these relationships was conducted using a linear mixed-effects model to evaluate whether the baseline association between distance from the fovea and MS varied by EZ score, including an interaction term between EZ score and distance from the fovea. All mixed-effects models included a random intercept to account for between-patient variability, with laterality nested within subject to control for intereye correlation when appropriate. A linear mixed-effects model was also utilized to evaluate for significant differences between genetic subtypes of RP in MS, as well as the proportion of loci with residual function in absence of visible EZ. The Tukey test was used to evaluate pairwise comparisons. All statistical analyses were conducted using the R programming language for statistical computation, version 4.4.0 (R Core Team 2024; R Foundation for Statistical Computing). *P* values <0.05 were considered statistically significant.

## Results

A total of 3388 test loci were graded for EZ structure and paired with their respective microperimetry sensitivity readings from baseline studies. Ultimately, 51 eyes from 31 patients were included in the baseline analysis. Nine additional overlays from 6 patients with follow-up studies available were also graded (597 test loci) and used in a longitudinal follow-up analysis. Mixed-effects models used baseline examinations only. A total of 91 eyes from 52 consecutive RP patients were initially reviewed. Of these, 20 were excluded because of the presence of cystoid macular edema, and 4 were excluded because of epiretinal membrane, which grossly altered macular anatomy on SD-OCT. Thirteen were excluded because of poor image quality or retinal structure abnormalities prohibiting accurate image overlay. Of those remaining, 3 were excluded because of high microperimetry fixation losses. The mean age of included patients was 39.4 (32.8, 46.0) years, with 41.9% being female. Mean best-corrected visual acuity was 0.166 (0.129, 0.203) logarithm of the minimum angle of resolution (Table 1). Specific genetic information was available for 38 eyes, with the largest genetic cluster consisting of 10 eyes with USH2A-associated RP, and the remaining eyes within clusters ranging in size from 1 to 6 eyes (Supplemental Table 1, available at www.ophthalmologyscience.org).

**Table 1:** Overall sohort and Eye-Level Demographic and Ocular Characteristics

Demographic and Ocular Characteristics	Overall (n = 31 Subjects, 51 Eyes)
Age (yrs)	39.4 (32.8–46.0)
Sex	
Female	13 (41.9%)
Male	18 (58.1%)
Race	
Asian	1 (3.2%)
Black or African American	3 (9.7%)
Other race or mixed race	8 (25.8%)
Unknown	2 (6.5%)
White	17 (54.8%)
Ethnicity	
Other Hispanic, Latino(a) or Spanish origin	1 (3.2%)
Not Hispanic, Latino(a), or Spanish origin	21 (67.7%)
Other Hispanic, Latino(a) or Spanish origin	8 (25.8%)
Unknown	1 (3.2%)
Mean sensitivity (dB), per eye	9.72 (7.32–12.12)
BCVA (logMar)	0.166 (0.129–0.203)

BCVA = best-corrected visual acuity; dB = decibels; logMAR = logarithm of the minimum angle of resolution.

To validate the grading system, an interobserver validation of all loci grades for all study eyes was first performed, yielding an agreement of 93.9% (3732 of 3985 loci), an unweighted Cohen kappa of 0.837 (*P* < 0.001), and a squared distance weighted kappa of 0.908 (*P* < 0.001), indicating high agreement. Test–retest also showed strong agreement with a Cohen kappa of 0.865 (*P* < 0.001).

Mean sensitivity in the cohort (Table 1) averaged 9.72 (7.32–12.12) dB per eye. Average sensitivity was also calculated for each EZ grade category, with grade 0 loci (68.5%) scoring on average 6.02 (4.06–7.98) dB, grade 1 loci (14.6%) scoring 18.36 (16.35–20.36) dB, and grade 2 loci (16.9%) scoring 20.90 (18.87–22.93) dB (Table 2). Mean sensitivities for each EZ grade category were significantly different from one another (all *P* < 0.001). The correlation between EZ grade and sensitivity was calculated, yielding a Spearman rank correlation of 0.65 (0.64–0.67), indicating a moderate to strong relationship. Diagnostic plots, including residuals versus fitted values, Q-Q plots, and histograms, were used to evaluate residuals in the mixed model. Residual evaluation indicated no significant deviations from normality and homoscedasticity and revealed no violations of mixed-effects model assumptions.

**Table 2:** Mean Sensitivity versus Ellipsoid Zone (EZ) Grade

	EZ Grade (n = 3388 Loci)			*P* Value		
	Grade 0 (n = 2322, 68.5%)	Grade 1 (n = 494, 14.6%)	Grade 2 (n = 572, 16.9%)	0 vs. 1	0 vs. 2	1 vs. 2
Mean sensitivity (dB)	6.02 (4.06–7.98)	18.36 (16.35–20.36)	20.90 (18.87–22.93)	**<0.001**	**<0.001**	**<0.001**

dB = decibels; EZ = ellipsoid zone; Grade 0 = EZ not visible; Grade 1 = EZ attenuated; Grade 2 = EZ normal.

Spearman rank correlation of mean sensitivity and EZ grade = 0.65 (0.64, 0.67), indicating a moderate to strong relationship.

Bold *P* values denote statistically significant data (*P* < 0.05).

**Figure 4:**
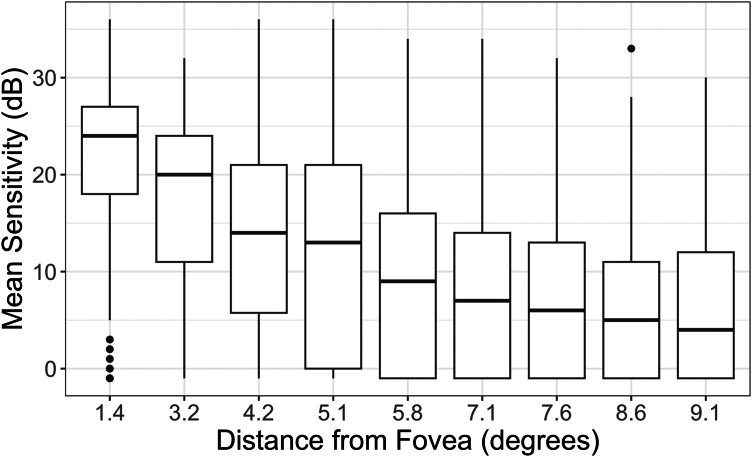
Mean sensitivity versus distance from the fovea. Box plot displaying median and IQR of mean sensitivity (decibels [dB]) per distance from the fovea. Pearson correlation between mean sensitivity and distance from the fovea is −0.41 (−0.43 to −0.39). IQR = interquartile range.

Sensitivity was next correlated with distance from the fovea, yielding a Pearson correlation coefficient of −0.41 (−0.43 to −0.39) (Fig 4). Further analysis evaluated the impact of eccentricity on sensitivity, and how it varied across EZ score classification. Moving away from the fovea, mean decrease in sensitivity per 1° increase in distance was significantly greater for EZ = 0 and EZ = 1 loci compared to EZ = 2 loci (0 vs. 2, *P* = 0.001; 1 vs. 2, *P* = 0.016) (Supplemental Figure 1, available at www.ophthalmologyscience.org).

Interestingly, in grade 0 loci, which were found to not have any EZ evident on SD-OCT, 57.5% had sensitivity scores >0 dB (we will abbreviate these loci “EZ = 0 S > 0”). Additionally, 4.0% of EZ = 0 loci had sensitivity scores that were markedly higher (≥20 dB) than would be expected for the EZ grading, demonstrating strong function in loci with no visible EZ.

Although microperimetry has been well studied and shown to be reliable, to understand whether the findings were due to nearby regions with EZ, we performed an additional nearest neighbor analysis that identified EZ = 0 S > 0 loci adjacent to loci with and without intact EZ. This was to determine what percentage of EZ = 0 S > 0 loci may be registering higher sensitivities because of EZ in neighboring loci. Fifty-nine percent of EZ = 0 S > 0 loci had no neighboring loci with visible EZ, and 41% had neighboring loci with some visible EZ. This suggests that many EZ = 0 S > 0 loci were located in regions with no visible EZ in surrounding areas, and that microperimetry sampling from nearby areas with functional EZ was unlikely.

To further validate our findings of discrepancies between EZ grading and microperimetry sensitivity scores at certain loci, we looked at our longitudinal data to ensure that these measurements were not errors at 1 timepoint. For the 6 patients with follow-up studies, a longitudinal analysis compared EZ = 0 S > 0 loci before and after follow-up to assess for consistency. Mean follow-up time was 1.49 (0.88, 2.11) years. Of these loci, 82.7% remained EZ = 0 S > 0 longitudinally, 13.3% had sensitivity fall below 1 dB, and 4.6% had EZ grade change (borderline 0 vs. 1 regions). For EZ = 0 loci with baseline sensitivity ≥20 dB, 75.0% fell below 25 dB over follow-up time, but 25.0% remained above 25 dB, supporting our finding that at certain loci there was a discrepancy between observable EZ on OCT and microperimetric function.

Lastly, to identify any non-EZ-like structural features at loci that might be associated with higher visual function, a manual review of SD-OCT images for EZ = 0 loci with sensitivities ≥20 dB was conducted. However, no unique structural features or patterns were observed at these loci (Supplemental Figure 2, available at www.ophthalmologyscience.org).

## Discussion

This study investigated the pointwise EZ structure–function relationship in a cohort of RP patients. This was done by overlaying NIR images from SD-OCT onto NIR images from MAIA microperimetry. This facilitated grading of the EZ structure at each of 68 microperimetry loci in the central 20° of the macula, thereby precisely correlating structure with function. As expected for RP patients, the average MS score (9.72 dB) was lower than normal (>25 dB, approximately),^17^ with sensitivity most preserved near the fovea. Relatively more intact EZ was also found near the fovea, as expected for classical RP.^1^^,^^18^ On average, sensitivity fell with less visible EZ (correlation = 0.65). Interestingly, however, nonzero sensitivity scores were frequently observed in grade 0 regions where no EZ was visible on SD-OCT. On average, grade 0 regions had a sensitivity of 6.02 dB and, in some cases, had sensitivities exceeding 20 dB.

Prior studies using SD-OCT and microperimetry have also investigated the EZ structure–function nonpointwise relationships in RP and found significant correlations. Asahina et al^21^ and Lad et al^22^ found MS declined with a smaller area of intact EZ. Asahina et al used the Nidek MP-3 microperimeter as well as the Humphrey field analyzer, finding a significant structure–function correlation with the MP-3. Similar to our study, Lad et al performed an OCT-MAIA overlay; however, this looked specifically at *USH2A*-associated RP and autosomal recessive RP. Mean sensitivity was also analyzed inside and outside of the area of ellipsoid zone rather than through a pointwise structure–function analysis. Funatsu et al^23^ did perform a pointwise analysis using the Nidek MP-3 system and an SD-OCT overlay with Image Filing Software NAVIS EX (Ver. 1.5.5, Nidek), finding significant correlations between declining outer retinal thickness and sensitivity; however, the EZ was not studied specifically. Interestingly, they also noted cases where retinal sensitivity was relatively preserved despite outer retinal thinning, suggesting lags may exist between structural and subsequent functional loss. Additional studies using standard automated perimetry and Humphrey field analyzer have also found significant correlations between EZ, OS, or outer retinal thickness and sensitivity.^11^^,^^24^, ^25^, ^26^ Microperimetry, however, may offer greater accuracy and repeatability compared with these modalities and has the added benefit of fixation tracking.^21^, ^22^, ^23^ Somewhat similar to our results, but using standard automated perimetry, Smith et al found a stronger global correlation between EZ width and sensitivity (expressed as hill of vision slope) and a weaker local relationship. This study used a partly manual alignment method to align the optic disc and the natural scotoma in the visual field because there was no standard automated perimetry NIR image for direct overlay, which would likely limit the accuracy of the overlay.^24^

Our findings of nonzero sensitivities in grade 0 regions are interesting given the importance of the EZ for phototransduction. However, RP structure–function incongruencies^23^^,^^24^^,^^27^^,^^28^ have been noted in the literature, particularly at the local level. Together this suggests the local relationship between the EZ and sensitivity may be more nuanced than structural loss translating directly into an immediately measurable functional loss, or perhaps that viable photoreceptors remain in the retina despite not being visible on OCT. Disorganized but partially functioning photoreceptors that have lost OS structure may manifest as weak or even lost NIR reflectance signals.^28^ It has also been suggested that relative preservation of function despite structural abnormalities may vary with different RP subtypes.^27^ Our cohort, however, was heterogeneous (Supplemental Table 1), and significant MS differences between genetic clusters with relatively small sample sizes (range: 1 to 10 eyes per cluster) were not found. Significant differences in prevalence of EZ = 0 S > 0 loci between clusters, as well as prevalence of EZ = 0 S ≥ 20 loci, were also not found across all pairwise comparisons. We also considered the possibility that pointwise sensitivity could be influenced by adjacent loci with intact EZ. Although this could theoretically help explain up to 41% of EZ = 0 S > 0 loci, it does not explain the remaining 59%. In terms of overall correlation between structure and function, Funatsu et al noted the correlation between sensitivity and outer retinal thickness was weaker further from the fovea, possibly due to floor effects.^23^ We also found this pattern to be true with the EZ (Supplemental Table 2, available at www.ophthalmologyscience.org), with the exception of a weaker correlation at 1.4°.

This study was limited by its retrospective nature with a relatively small sample size of cases that fit our strict inclusion and exclusion criteria. Despite this, there were a large number of graded structure–function loci pairs (3985). There is also a possibility that there may be neighboring EZ beyond the narrow band of the cross-sectional SD-OCT B-scan, as there was 1 scan per locus and microperimetry loci were separated by 576 μm. Additionally, although we found that there were loci with visual function on repeat testing that did not demonstrate visible EZ on OCT, there is a possibility that other outer retinal layers may be providing a response. Ongoing studies aim to analyze the correlation of outer retinal layers, including the outer nuclear layer, by segmentation, to understand their relationship to visual function.

In conclusion, our group conducted a pointwise structure–function analysis of the EZ in RP using SD-OCT imaging with microperimetry overlay. The study identified photoreceptor structure–function incongruencies that may be overlooked by averaging across the visual field globally. A precise explanation for preserved photoreceptor function in the absence of visibly intact EZ remains unknown and warrants further investigation, particularly as the EZ continues to be used as a biomarker in RP studies. Concealed functional photoreceptors, not identifiable by structural imaging, could be a target for therapies to preserve vision.

## Supplementary Data

SupplementaryFigure 2

## References

[1] Tee J.J.L., Carroll J., Webster A.R., Michaelides M. (2017). Quantitative analysis of retinal structure using spectral-domain optical coherence tomography in RPGR-associated retinopathy. Am J Ophthalmol.

[2] Nguyen X.T.A., Moekotte L., Plomp A.S. (2023). Retinitis pigmentosa: current clinical management and emerging therapies. Int J Mol Sci.

[3] Oh R., Bae K., Yoon C.K. (2024). Quantitative microvascular analysis in different stages of retinitis pigmentosa using optical coherence tomography angiography. Sci Rep.

[4] Weleber R.G., Gregory-Evans K. (2006). Retinitis pigmentosa and allied disorders. Med Retina.

[5] Cross N., van Steen C., Zegaoui Y. (2022). Retinitis pigmentosa: burden of disease and current unmet needs. Clin Ophthalmol Auckl NZ.

[6] Prem Senthil M., Khadka J., Pesudovs K. (2017). Seeing through their eyes: lived experiences of people with retinitis pigmentosa. Eye.

[7] Fischer M.D., Simonelli F., Sahni J. (2024). Real-world safety and effectiveness of voretigene neparvovec: results up to 2 Years from the prospective, registry-based PERCEIVE study. Biomolecules.

[8] Hernández-Juárez J., Rodríguez-Uribe G., Borooah S. (2021). Toward the treatment of inherited diseases of the retina using CRISPR-based gene editing. Front Med.

[9] Zada M., Cornish E.E., Fraser C.L. (2021). Natural history and clinical biomarkers of progression in X-linked retinitis pigmentosa: a systematic review. Acta Ophthalmol (Copenh).

[10] Birch D.G., Locke K.G., Felius J. (2015). Rates of decline in fdOCT defined regions of the visual field in patients with RPGR-mediated X-linked retinitis pigmentosa (XLRP). Ophthalmology.

[11] Birch D.G., Locke K.G., Wen Y. (2013). Spectral-domain optical coherence tomography measures of outer segment layer progression in patients with X-linked retinitis pigmentosa. JAMA Ophthalmol.

[12] Iga Y., Hasegawa T., Ikeda H.O. (2023). Progression of retinitis pigmentosa on static perimetry, optical coherence tomography, and fundus autofluorescence. Sci Rep.

[13] Berni A., Arrigo A., Bianco L. (2024). New insights in the multimodal imaging of retinitis pigmentosa. Eur J Ophthalmol.

[14] Yassin S.H., Wang Y., Freeman W.R. (2023). Efficacy and accuracy of artificial intelligence to overlay multimodal images from different optical instruments in patients with retinitis pigmentosa. Clin Exp Ophthalmol.

[15] MAIA (2019). Macular Integrity Assessment Operating Manual.

[16] (2023). Heidelberg Engineering User Manual, Software version 7.0. Spectralis Product Family, Document No D4000020-001 US.AE23.

[17] Charng J., Sanfilippo P.G., Attia M.S. (2020). Interpreting MAIA microperimetry using age- and retinal loci-specific reference thresholds. Transl Vis Sci Technol.

[18] Barteselli G., Bartsch D.U., Viola F. (2013). Accuracy of the Heidelberg Spectralis in the alignment between near-infrared image and tomographic scan in a model eye: a multicenter study. Am J Ophthalmol.

[19] DeTone D., Malisiewicz T., Rabinovich A. (2018). 2018 IEEE/CVF Conference on Computer Vision and Pattern Recognition Workshops (CVPRW).

[20] Aizawa S., Mitamura Y., Baba T. (2009). Correlation between visual function and photoreceptor inner/outer segment junction in patients with retinitis pigmentosa. Eye.

[21] Asahina Y., Kitano M., Hashimoto Y. (2017). The structure-function relationship measured with optical coherence tomography and a microperimeter with auto-tracking: the MP-3, in patients with retinitis pigmentosa. Sci Rep.

[22] Lad E.M., Duncan J.L., Liang W. (2022). Baseline microperimetry and OCT in the RUSH2A study: structure-function association and correlation with disease severity. Am J Ophthalmol.

[23] Funatsu J., Murakami Y., Nakatake S. (2019). Direct comparison of retinal structure and function in retinitis pigmentosa by co-registering microperimetry and optical coherence tomography. PLoS One.

[24] Smith T.B., Parker M., Steinkamp P.N. (2016). Structure-function modeling of optical coherence tomography and standard automated perimetry in the retina of patients with autosomal dominant retinitis pigmentosa. PLoS One.

[25] Hood D.C., Ramachandran R., Holopigian K. (2011). Method for deriving visual field boundaries from OCT scans of patients with retinitis pigmentosa. Biomed Opt Express.

[26] Rangaswamy N.V., Patel H.M., Locke K.G. (2010). A comparison of visual field sensitivity to photoreceptor thickness in retinitis pigmentosa. Invest Ophthalmol Vis Sci.

[27] Micevych P.S., Wong J., Zhou H. (2023). Cone structure and function in RPGR- and USH2A-associated retinal degeneration. Am J Ophthalmol.

[28] Buckley T.M.W., Jolly J.K., Josan A.S. (2021). Clinical applications of microperimetry in RPGR-related retinitis pigmentosa: a review. Acta Ophthalmol (Copenh).

