# Supplementary material for: Pointwise Structure–Function Analysis of the Ellipsoid Zone in Retinitis Pigmentosa Using an Artificial Intelligence-Assisted OCT and Microperimetry Overlay
Source: Ophthalmol Sci. 2025 Jul 21;5(6):100889. doi: 10.1016/j.xops.2025.100889 (PMC12446769; doi:10.1016/j.xops.2025.100889)
Supplement: SupplementaryFigure 2 [file mmc1.pdf]

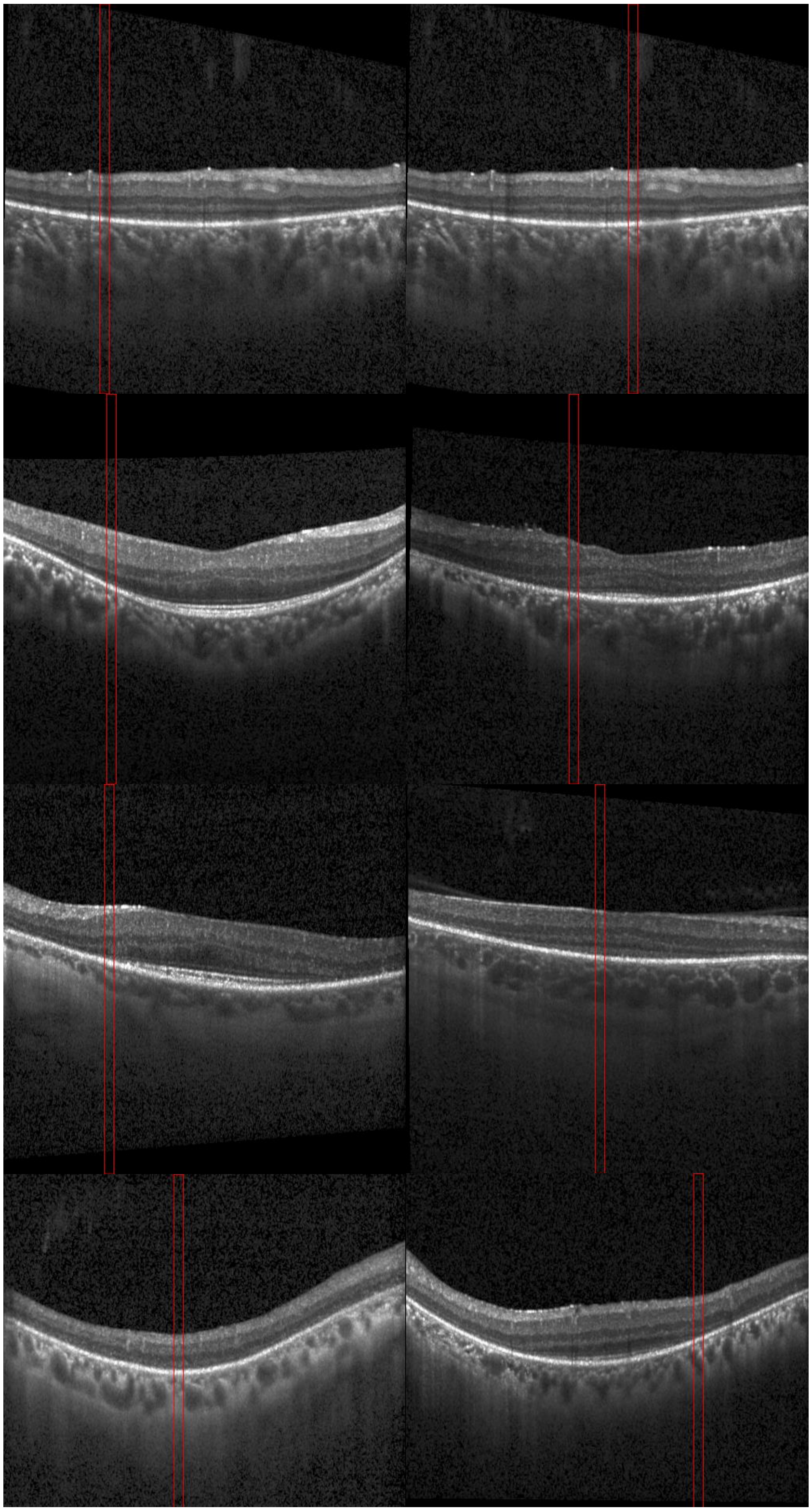

**Supplemental Figure 2. ‘EZ=0 S≥20 dB’ loci example SD-OCT images**
Representative SD-OCT image set of loci with no visible ellipsoid zone (EZ=0) and sensitivity scores ≥20 decibels (dB). No unique retinal features or patterns were observed at these loci.
