# Supplementary material for: Pointwise Structure–Function Analysis of the Ellipsoid Zone in Retinitis Pigmentosa Using an Artificial Intelligence-Assisted OCT and Microperimetry Overlay
Source: Ophthalmol Sci. 2025 Jul 21;5(6):100889. doi: 10.1016/j.xops.2025.100889 (PMC12446769; doi:10.1016/j.xops.2025.100889)
Supplement: Supplementary Table 2 [file mmc2.pdf]

Degrees from fovea	EZ Grade vs. Sensitivity Correlation
Degrees	Correlation
1.4	0.53 (0.42, 0.63)
3.2	0.64 (0.57, 0.70)
4.2	0.63 (0.53, 0.71)
5.1	0.67 (0.60, 0.72)
5.8	0.66 (0.59, 0.72)
7.1	0.58 (0.52, 0.64)
7.6	0.56 (0.49, 0.63)
8.6	0.52 (0.44, 0.60)

Supplemental Table 2.
Ellipsoid zone versus sensitivity correlations, stratified by distance from the fovea
Spearman’s rank correlation and 95% confidence interval estimates evaluating the relationship between microperimetry sensitivity and ellipsoid zone (EZ) grade, per distance from the fovea in degrees.
