# Supplementary material for: Pointwise Structure–Function Analysis of the Ellipsoid Zone in Retinitis Pigmentosa Using an Artificial Intelligence-Assisted OCT and Microperimetry Overlay
Source: Ophthalmol Sci. 2025 Jul 21;5(6):100889. doi: 10.1016/j.xops.2025.100889 (PMC12446769; doi:10.1016/j.xops.2025.100889)
Supplement: Supplementary Figure 1 [file mmc3.pdf]

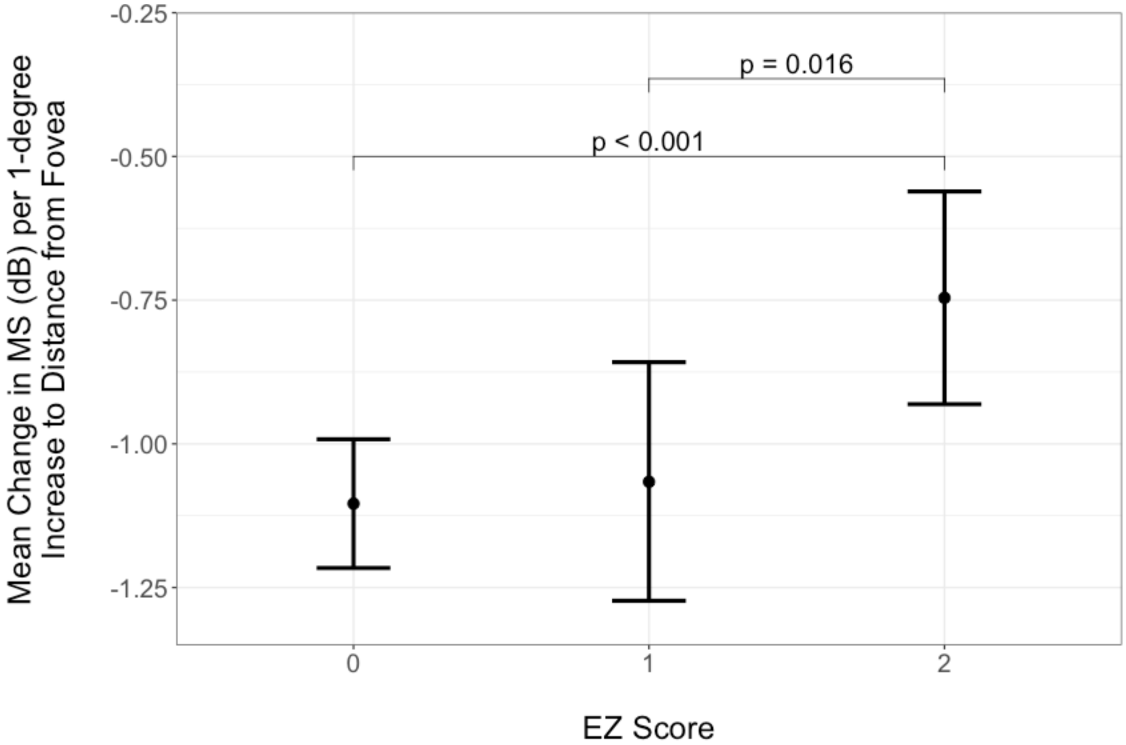

Supplemental Figure 1. Linear mixed-effects model predicting mean sensitivity (MS) using ellipsoid zone (EZ) score, degrees from the fovea, and an interaction term between EZ and degrees.
