# Supplementary material for: Pointwise Structure–Function Analysis of the Ellipsoid Zone in Retinitis Pigmentosa Using an Artificial Intelligence-Assisted OCT and Microperimetry Overlay
Source: Ophthalmol Sci. 2025 Jul 21;5(6):100889. doi: 10.1016/j.xops.2025.100889 (PMC12446769; doi:10.1016/j.xops.2025.100889)
Supplement: Supplementary Table 1 [file mmc4.pdf]

Supplemental Table 1. Cohort RP subtypes and genetic characteristics

RP subtype and genetic testing results for 22 of 31 patients where this information was available. Patients with no known subtype or genetic testing results not shown.

Genetic Subtype	Number of Eyes	Mutation Result	Inheritance Pattern
EYS	4	EYS: c.5928-2A>G (Splice acceptor), homozygous, pathogenic	AR
		EYS: c.5928-2A>G (Splice acceptor), homozygous, pathogenic	AR
		EYS: c.6557G>A (p.Gly2186Glu), heterozygous, pathogenic and EYS: c.1083_1108del (p.Phe362Serfs*5), heterozygous, pathogenic; phase of EYS variants unknown	AR
		EYS: c.6557G>A (p.Gly2186Glu), heterozygous, pathogenic and EYS: c.1083_1108del (p.Phe362Serfs*5), heterozygous, pathogenic; phase of EYS variants unknown	AR
ARSG Usher	1	ARSG c.1326del (p.Ser443Alafs*12) heterozygous pathogenic	AR
CERKL	1	CERKL: c.847C>T (p.Arg283*), homozygous, pathogenic	AR
CNGB1	2	CNGB1: c.3139_3142dup (p.Ala1048Glyfs*13), heterozygous, pathogenic and CNGB1: c.2957A>T (p.Asn986Ile), heterozygous, pathogenic; phase of CNGB1 variants unknown	AR
		CNGB1: c.3139_3142dup (p.Ala1048Glyfs*13), heterozygous, pathogenic and CNGB1: c.2957A>T (p.Asn986Ile), heterozygous, pathogenic; phase of CNGB1 variants unknown	AR
PDE6A	2	PDE6A: c.475-2A>G, heterozygous, likely pathogenic and PDE6A: c.84C>A (p.Tyr28*), heterozygous, likely pathogenic; phase of PDE6A variants unknown	AR
		PDE6A: c.475-2A>G, heterozygous, likely pathogenic and PDE6A: c.84C>A (p.Tyr28*), heterozygous, likely pathogenic; phase of PDE6A variants unknown	AR
POMGNT-1	2	POMGNT1: c.385C>T (p.Arg129Trp), heterozygous, pathogenic and POMGNT1: c.1099C>T (p.Arg367Cys), heterozygous, likely pathogenic; POMGNT1 variants confirmed in trans	AR
		POMGNT1: c.385C>T (p.Arg129Trp), heterozygous, pathogenic and POMGNT1: c.1099C>T (p.Arg367Cys), heterozygous, likely pathogenic; POMGNT1 variants confirmed in trans	AR
PRPF31	3	PRPF31: Deletion (Exon 14), heterozygous, likely pathogenic	AD

		PRPF31: c.1110_1117del (p.Ile371Alafs*101), heterozygous, likely pathogenic	AD
		PRPF31: c.1110_1117del (p.Ile371Alafs*101), heterozygous, likely pathogenic	AD
RHO	2	RHO:c.563G>A (p.Gly188Glu), heterozygous, pathogenic	AD
		RHO: c.1039C>A (p.Pro347Thr), heterozygous, pathogenic	AD
RP1	6	RP1: c.2626A>T (p.Lys876*), heterozygous, pathogenic and RP1: c.458dup (p.Arg154Thrfs*75), heterozygous, pathogenic; RP1 variants confirmed in trans	AR
		RP1: c.2626A>T (p.Lys876*), heterozygous, pathogenic and RP1: c.458dup (p.Arg154Thrfs*75), heterozygous, pathogenic; RP1 variants confirmed in trans	AR
		RP1: c.2374A>T (p.Lys792*), heterozygous, pathogenic	AD
		RP1: c.2374A>T (p.Lys792*), heterozygous, pathogenic	AD
		RP1: c.2197delA (p.Ser734Valfs*4), heterozygous, likely pathogenic; confirmed paternally inherited	AD
		RP1: c.2197delA (p.Ser734Valfs*4), heterozygous, likely pathogenic; confirmed paternally inherited	AD
RPGR	3	RPGR: c.2405_2406del (p.Glu802Glyfs*32), hemizygous, pathogenic	X-linked
		RPGR: c.905G>A, p.(Cys302Tyr), hemizygous, pathogenic.	X-linked
		RPGR: c.905G>A, p.(Cys302Tyr), hemizygous, pathogenic.	X-linked
SCAPER	2	SCAPER: c.3781del (p.Val1261Serfs*26), heterozygous, likely pathogenic and SCAPER: c.868_869del (p.Glu290Serfs*7), heterozygous, likely pathogenic; SCAPER variants confirmed in trans	AR
		SCAPER: c.3781del (p.Val1261Serfs*26), heterozygous, likely pathogenic and SCAPER: c.868_869del (p.Glu290Serfs*7), heterozygous, likely pathogenic; SCAPER variants confirmed in trans	AR
USH2A	10	USH2A: c.11864G>A (p.Trp3955*), heterozygous, pathogenic and USH2A c.4124C>T (p.Ser1375Leu), heterozygous, likely pathogenic; USH2A variants confirmed in trans	AR
		USH2A: c.11864G>A (p.Trp3955*), heterozygous, pathogenic and USH2A c.4124C>T (p.Ser1375Leu), heterozygous, likely pathogenic; USH2A variants confirmed in trans	AR
		USH2A: c.14219C>A (p.Ala4740Asp), heterozygous, pathogenic and USH2A c.7713_7724del (p.Gly2572_Tyr2575del), heterozygous, variant of uncertain significance; USH2A variants confirmed in trans	AR

		USH2A: c.14219C>A (p.Ala4740Asp), heterozygous, pathogenic and USH2A c.7713_7724del (p.Gly2572_Tyr2575del), heterozygous, variant of uncertain significance; USH2A variants confirmed in trans	AR
		USH2A: c.1859G>T (p.Cys620Phe), heterozygous, pathogenic and USH2A: c.13812-1G>A (Splice acceptor), heterozygous, pathogenic; USH2A variants confirmed in trans	AR
		USH2A: c.1859G>T (p.Cys620Phe), heterozygous, pathogenic and USH2A: c.13812-1G>A (Splice acceptor), heterozygous, pathogenic; USH2A variants confirmed in trans	AR
		USH2A: c.5836C>T (p.Arg1946Ter), heterozygous, pathogenic and USH2A: c.2299delG, (p.Glu767Serfs*21), heterozygous, pathogenic; phase of USH2A variants unknown	AR
		USH2A: c.956G>A (p.Cys319Tyr), homozygous, pathogenic	AR
		USH2A: c.2276G>T (p.Cys759Phe), heterozygous pathogenic and USH2A: c.5857+2T>C (Splice donor), heterozygous pathogenic; USH2A variants confirmed in trans	AR
		USH2A: c.2276G>T (p.Cys759Phe), heterozygous pathogenic and USH2A: c.5857+2T>C (Splice donor), heterozygous pathogenic; USH2A variants confirmed in trans	AR
